# Exercise has differential cardiometabolic effects in male and female mice on a high‐fat diet

**DOI:** 10.14814/phy2.70656

**Published:** 2026-01-28

**Authors:** L. E. Watson, M. Annandale, C. L. MacRae, J. Bai, J. Dayaram, N. Burgess, C. Puliuvea, C. P. Hedges, R. F. D'Souza, T. L. Merry, K. M. Mellor

**Affiliations:** ^1^ Department of Nutrition University of Auckland Auckland New Zealand; ^2^ Department of Physiology University of Auckland Auckland New Zealand; ^3^ Maurice Wilkins Centre of Research Excellence (CoRE) Auckland New Zealand; ^4^ Department of Molecular Medicine and Pathology University of Auckland Auckland New Zealand

**Keywords:** cardiac dysfunction, inflammation, metabolic disease, sex differences

## Abstract

Sex differences in the metabolic and anti‐inflammatory effects of exercise have been reported, but whether males and females exhibit a differential response to exercise in a setting of cardiometabolic disease is unknown. The objective of this study was to investigate the glucose handling, adipose and cardiac effects of voluntary exercise in male and female mice in a cardiometabolic disease setting induced by a high‐fat diet (HFD). The extent of exercise tolerance improvement was similar between HFD male and HFD female mice with running wheel access, despite greater daily running distances in female HFD mice. Exercise attenuated HFD‐induced increased body and fat mass in females but had no effect in males. A slight improvement in insulin tolerance was observed in HFD males only. The anti‐inflammatory effects of exercise were evident in both HFD males and HFD females, but the inflammatory cell types and tissue depots involved were sex‐specific. Cardiac diastolic function was improved with exercise in HFD females but not HFD males. Surprisingly, cardiomyocyte dimensions increased with exercise in HFD females and decreased with exercise in HFD males. This study provides the first evidence that the cardiometabolic effects of exercise are differentially elicited in males and females in a metabolic disease setting.

## 1 INTRODUCTION

Cardiometabolic disease is a collective term for metabolic abnormalities such as obesity, insulin resistance, impaired glucose homeostasis, dyslipidaemia, chronic inflammation, and hypertension (Kirk & Klein, 2009). The cardiac impact of these conditions includes cardiomyopathy, myocardial infarction, and heart failure (Flora & Nayak, 2019; Tune et al., 2017). Biological sex is known to impact the progression, prevalence, and outcomes of cardiometabolic disease (Regitz‐Zagrosek & Gebhard, 2023). Premenopausal women typically exhibit more favorable metabolic health profiles compared with men, exhibiting a lower percentage of visceral fat, enhanced skeletal muscle glucose uptake, and a delayed onset of cardiovascular disease and type 2 diabetes by about 10 years (Chella Krishnan et al., 2018; Nuutila et al., 1995). Experimentally, female mice appear to be relatively protected from the impact of a high‐fat diet, with a lower extent of weight gain, insulin resistance and inflammation than male mice (Braga Tibaes et al., 2024; Casimiro et al., 2021; Huang et al., 2020; Pettersson et al., 2012).

Regular physical exercise is beneficial for the prevention and treatment of cardiometabolic disease. Exercise is linked with enhancing fatty acid oxidation, promoting muscular hypertrophy and excess fat loss, improving glucose homeostasis and serum lipid profile, and improving cardiac function in patients with cardiovascular disease (Pinckard et al., 2019; Warburton et al., 2006). Additionally, exercise training has been demonstrated to inhibit adipose tissue inflammation in diet‐induced obese mice (Kawanishi et al., 2010). Lipid utilization with exercise is most prominent with low intensity, long duration exercise modalities, where fatty acids are liberated from adipose tissue and intramuscular triglyceride stores. This effect of exercise on lipid utilization has been observed in mice and humans, despite evidence that mice have a greater reliance on fatty acid oxidation overall (Axsom et al., 2024). Sex differences have been observed in physiological responses to exercise training in healthy human subjects, with females exhibiting greater fatty acid oxidation in skeletal muscle, and males exhibiting greater maximal oxygen uptake (Landen et al., 2023; Tarnopolsky, 2000). Cardiac function is also altered by biological sex in exercise, with males exhibiting greater cardiac output than females in response to acute exercise (Wheatley et al., 2014) and greater gains in maximal oxygen consumption in response to endurance training (Diaz‐Canestro & Montero, 2019; Thomas et al., 2023). A recent study of >400 k healthy adults in the United States revealed an association between surveyed regular physical activity and decreased risk of cardiovascular mortality that was more pronounced in women than in men (24% vs. 15% lower risk, respectively) (Ji et al., 2024). Collectively these studies in healthy settings suggest that females may gain greater cardiometabolic benefit from regular exercise compared with males. The underlying mechanisms have not been fully elucidated, and whether exercise benefit is sexually dimorphic in a setting of cardiometabolic disease has not been established.

Therefore, the objective of this study was to investigate the glucose handling, adipose and cardiac effects of voluntary exercise (running wheel) in male and female mice in a setting of cardiometabolic disease. A high‐fat diet mouse model was employed to recapitulate the clinical scenario of obesity and cardiometabolic dysfunction. The effects of voluntary running wheel exercise on weight gain and body composition, glucose homeostasis, adipose tissue inflammation, and cardiac function and morphology in HFD mice were examined.

## 2 RESULTS

### 2.1 Voluntary exercise attenuates HFD‐induced weight gain in female but not male mice

**FIGURE 1:**
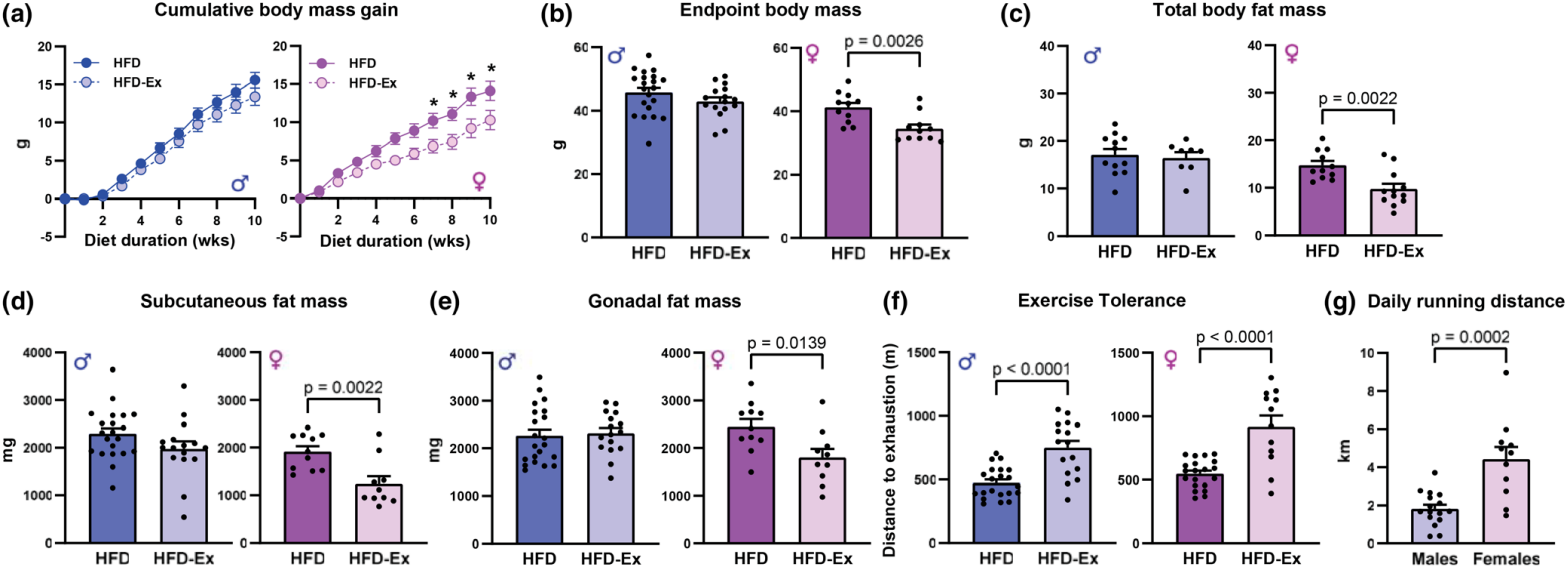
Voluntary exercise attenuated HFD‐induced weight gain in female, but not male mice. (a) Cumulative body mass gain of male and female HFD and HFD‐Ex mice over 10 weeks of HFD feeding. Female week 7, *p* = 0.026; week 8, *p* = 0.011; week 9, *p* = 0.001; week 10, *p* = 0.003 HFD‐Ex versus HFD. (b) Endpoint body mass of male and female HFD and HFD‐Ex mice. (c) Total body fat mass of male and female HFD and HFD‐Ex mice. (d) Subcutaneous fat mass of male and female HFD and HFD‐Ex mice. (e) Gonadal fat mass of male and female HFD and HFD‐Ex mice. (f) Exercise tolerance (distance ran to exhaustion on treadmill) of male and female HFD and HFD‐Ex mice. (g) Average daily running distance for male and female HFD‐Ex mice. Data are presented as individual values, mean ± SEM. Data were analyzed by 2‐way ANOVA with repeated measures and Bonferroni post hoc tests (a) or Student's *t*‐test (b–g).

During 10 weeks of consuming a HFD consisting of 60% calories from fat, high‐fat diet exercised (HFD‐Ex) female mice exhibited significantly attenuated cumulative body mass gain compared with HFD females (exercise factor effect *p* = 0.015). Whereas there were no significant differences in cumulative body mass gain between HFD males and HFD‐Ex males (Figure 1a, *p* = 0.22). At study endpoint, HFD‐Ex female mice had significantly smaller total body mass on average when compared with HFD females (Figure 1b, *p* = 0.0026), while no difference was observed between HFD males and HFD‐Ex males (Figure 1b, *p* = 0.17). This difference in body mass gain in females appeared to be driven by changes in body fat, with HFD‐Ex females exhibiting decreased total body fat mass (Figure 1c, *p* = 0.015), and a decrease in both subcutaneous (Figure 1d, *p* = 0.0022) and gonadal (Figure 1e, *p* = 0.014) fat pad mass. Lean mass was unchanged in females and slightly increased in males (Table S2). Exercise tolerance was increased similarly by voluntary wheel running in both HFD‐Ex males (*p* < 0.0001) and HFD‐Ex females (*p* < 0.0001, Figure 1f), despite HFD‐Ex females running greater average daily distances than HFD‐Ex males (Figure 1g, *p* = 0.0002). Daily running distance declined over the 12‐week diet intervention period for both male and female mice, yet HFD‐Ex females exhibited greater running distance than males at all timepoints (Figure S1a). Analysis of the relationship between body mass or fat mass with running distance did not reveal any significant correlations for male or female HFD‐Ex mice (Figure S1b,c). Collectively, these data suggest that higher running distances in females are associated with attenuation of HFD‐induced body and fat mass gain.

### 2.2 The anti‐inflammatory effect of exercise is differentially instigated in male and female HFD mice

**FIGURE 2:**
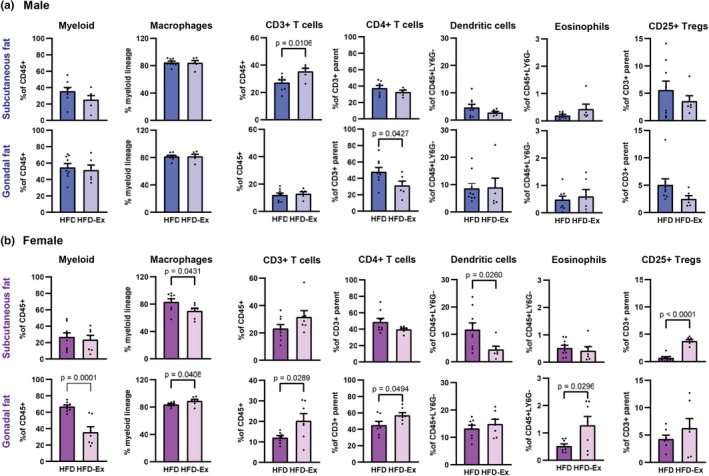
The anti‐inflammatory response to exercise is differentially instigated in male and female HFD mice. (a) Adipose immune cell populations in subcutaneous and gonadal fat pads of male HFD and HFD‐Ex mice following 10 weeks of HFD feeding. (b) Adipose immune cell populations in subcutaneous and gonadal fat pads of female HFD and HFD‐Ex mice following 10 weeks of HFD feeding. Data are presented as individual values ± SEM. Data were analyzed by Student's *t*‐test.

Following the observation that voluntary exercise attenuated the expansion of both subcutaneous and gonadal fat pads in female but not male mice on the HFD, we sought to determine if this resulted in greater improvements in adipose tissue inflammation, which is a hallmark of diet‐induced obesity (Kawai et al., 2021). In male HFD mice, exercise elicited an anti‐inflammatory effect in the subcutaneous and gonadal fat pads, as evidenced by an exercise‐induced reduction in CD4+ T cells in the gonadal fat (Figure 2a, *p* = 0.043). However, an anomalous pro‐inflammatory action was observed in the subcutaneous fat, evidenced by an expansion of CD3+ T cells (Figure 2a, *p* = 0.011)—as T cell infiltration/expansion in adipose tissue is a hallmark of inflammation associated with diet‐induced obesity (Liao et al., 2024). In female mice, exercise decreased macrophages and dendritic cells (Figure 2b, *p* = 0.043 and *p* = 0.026, respectively) and increased CD25+ regulatory T cells (Figure 2b, *p* < 0.0001) in the subcutaneous fat. In the gonadal fat, an anti‐inflammatory effect of exercise response was marked by a decrease in myeloid cells (Figure 2b, *p* = 0.0001) and increased eosinophils (*p* = 0.030). Anomalous pro‐inflammatory actions in gonadal fat were also observed, as evidenced by an increase in macrophages, CD3+ and CD4+ T cells (Figure 2b, *p* = 0.041, 0.029, and 0.049, respectively). Although some inconsistent observations were evident, both males and females exhibited anti‐inflammatory effects of exercise; however, sex‐specific differences in inflammatory cell responses were apparent.

### 2.3 Voluntary exercise attenuated HFD‐induced insulin intolerance in male but not female mice

**FIGURE 3:**
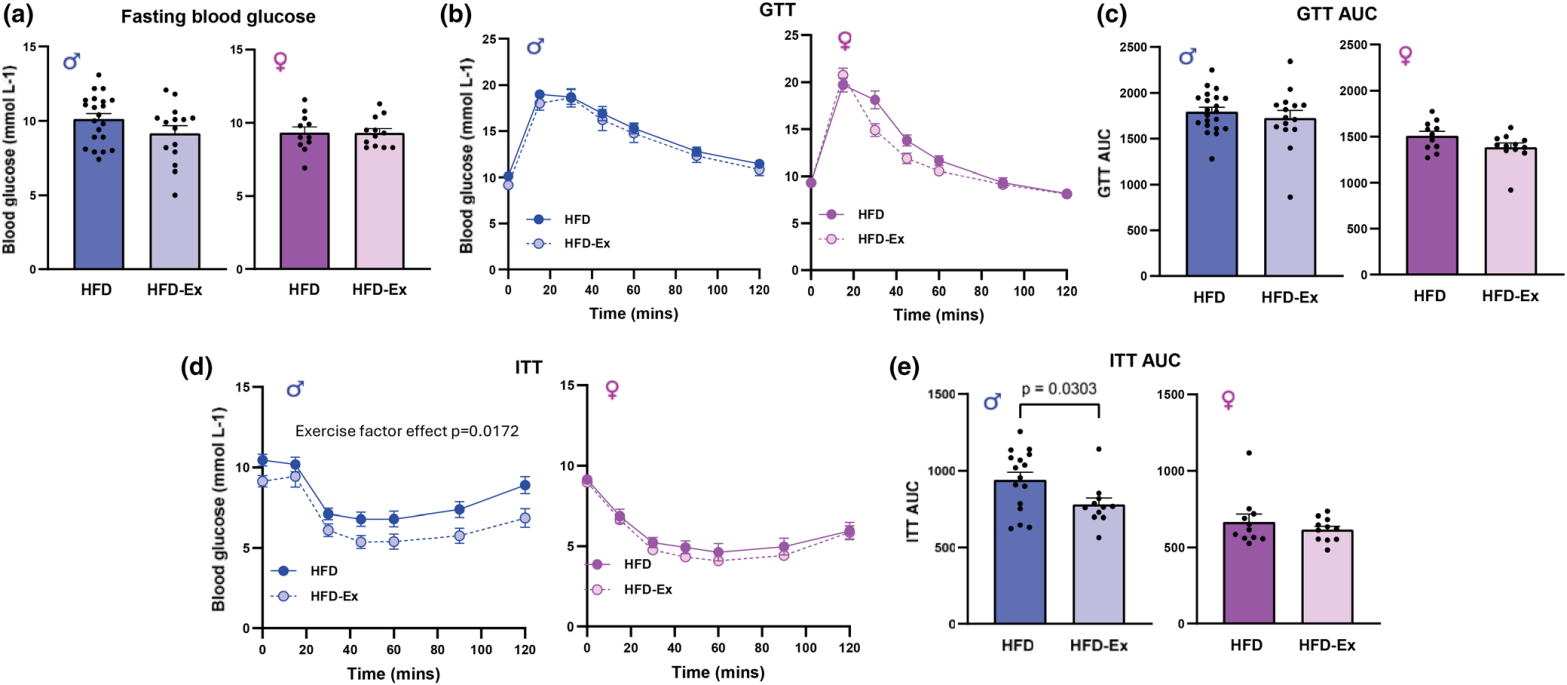
Voluntary exercise attenuated HFD‐induced insulin resistance in male but not female mice. (a) Blood glucose following a 6‐h fast in male and female HFD and HFD‐Ex mice following 10 weeks of HFD feeding. (b) Glucose tolerance test (GTT) in 6‐h fasted male and female HFD and HFD‐Ex mice. (c) GTT area‐under‐curve in 6‐h fasted male and female HFD and HFD‐Ex mice. (d) Insulin tolerance test (ITT) in 6‐h fasted male and female HFD and HFD‐Ex mice. (e) ITT area‐under‐curve in 6‐h fasted male and female HFD and HFD‐Ex mice. Data are presented as individual values ± SEM. Data were analyzed by 2‐way ANOVA with repeated measures (b and d) or Student's *t*‐test (a, c, and e).

Chronic adipose tissue inflammation in diet‐induced obesity is closely linked to systemic metabolic dysfunction. As it has been previously observed that HFD feeding over 10 weeks impairs glucose and insulin tolerance in both male and female mice (Friemel et al., 2017; Varghese et al., 2020) we chose to examine sex differences in blood glucose control following the voluntary exercise intervention. Following a 6 h fast, HFD and HFD‐Ex males and HFD and HFD‐Ex females had similar fasting blood glucose concentrations (Figure 3a, *p* = 0.12 and 0.97, respectively). The area under the glucose tolerance curve was decreased with exercise in HFD‐Ex females, but this effect did not reach significance (*p* = 0.075, Figure 3c). No effect of exercise on glucose tolerance was observed in males (*p* = 0.46, Figure 3b,c). An improvement in insulin tolerance was observed in HFD males (*p* = 0.03) but not females (*p* = 0.37) with exercise (Figure 3d,e). These data suggest that, while voluntary exercise does not impact glucose tolerance in HFD‐fed male and female mice, insulin sensitivity may be improved by exercise in males.

### 2.4 Voluntary exercise alters cardiac function in HFD female, but not HFD male mice

**FIGURE 4:**
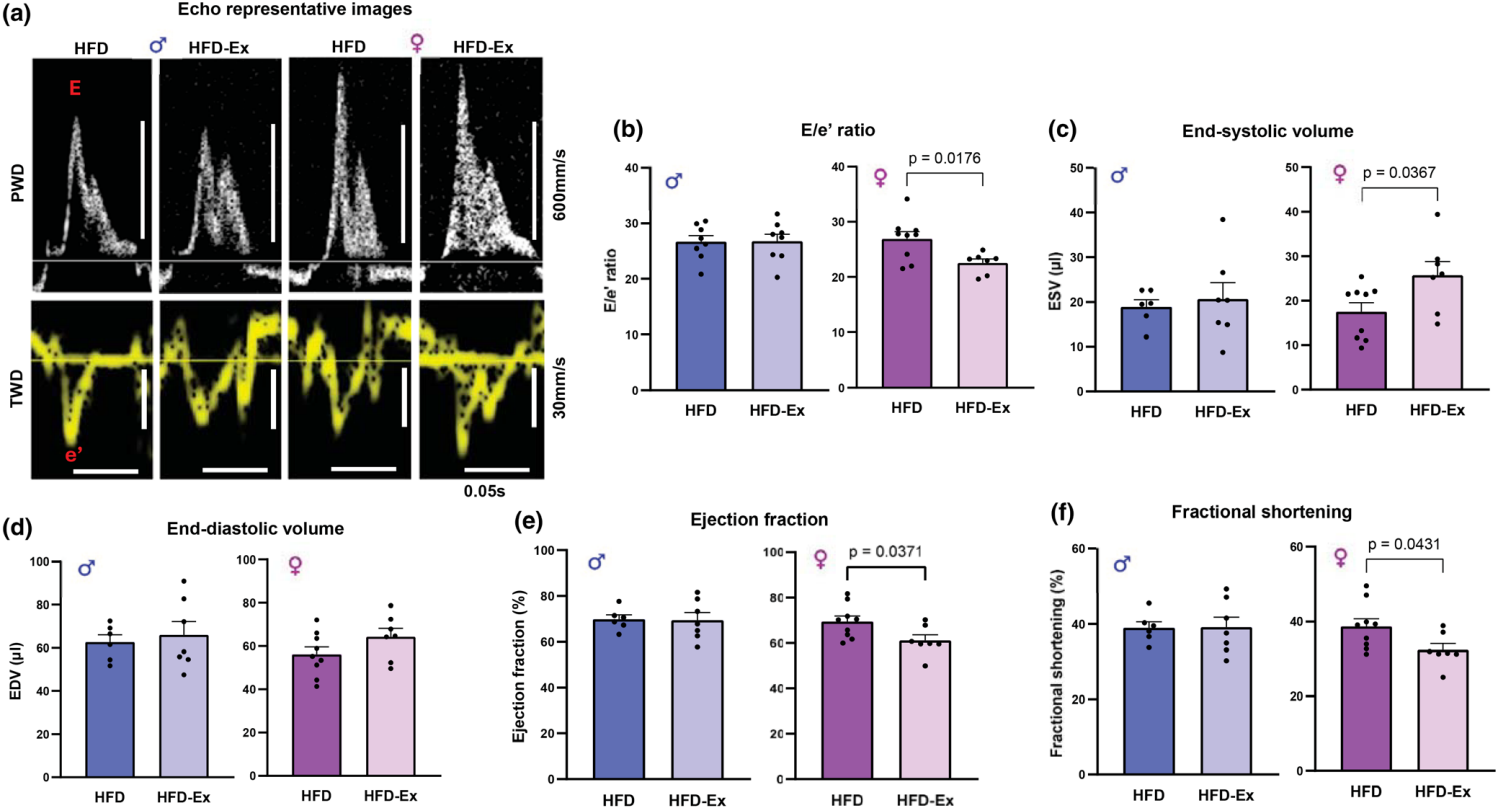
Voluntary exercise improves diastolic function in female, but not male mice. (a) Representative images of Doppler ultrasounds of male and female HFD and HFD‐Ex mice hearts following 10 weeks of HFD feeding (b) E/e′ ratios of male and female HFD and HFD‐Ex mice. (c) End‐systolic volume in male and female HFD and HFD‐Ex mice hearts. (d) End‐diastolic volume in male and female HFD and HFD‐Ex mice hearts. (e) Ejection fraction in male and female HFD and HFD‐Ex mice hearts. (f) Fractional shortening in male and female HFD and HFD‐Ex mice hearts. Data are presented as individual values ± SEM. Data were analyzed by Student's *t*‐test.

Previous work from our group has demonstrated that HFD feeding results in impairment in cardiac diastolic function with preserved systolic function in mice (Daniels et al., 2023). As beneficial cardiac outcomes following exercise interventions are commonly reported (Pinckard et al., 2019); we then investigated whether cardiac function was improved with exercise in our model of HFD‐fed male and female mice. HFD‐Ex female mice exhibited a significant reduction in the ratio of early mitral valve blood flow (E wave) to early mitral annulus tissue velocity (e′ wave) (E/e′ ratio, Figure 4b, *p* = 0.018) compared with HFD females, indicating improved diastolic function. No change in E/e′ ratio was observed between HFD and HFD‐Ex males (Figure 4b, *p* = 0.95). End‐systolic volume was increased in HFD‐Ex females compared with HFD females (Figure 4c, *p* = 0.037) while males remained unaffected (*p* = 0.68). No effect of exercise on end‐diastolic volume was observed in HFD‐Ex mice of either sex (Figure 4d, *p* = 0.65 (males) and *p* = 0.14 (females)). Interestingly, ejection fraction and fractional shortening were slightly decreased in HFD‐Ex females compared with HFD females (Figure 4e, *p* = 0.037 and Figure 4f, *p* = 0.043). Exercise did not elicit any differences in these variables in the male mice (Figure 4e, *p* = 0.93 and Figure 4f, *p* = 0.97). Cardiac output and heart rate were unchanged with exercise in male and female HFD mice (Table S1). These data suggest that while cardiac function is unaffected by exercise in male HFD‐fed mice, exercise elicits beneficial effects on diastolic function in HFD‐fed female mice. Some minor decreases in measures of systolic function were observed in HFD‐fed female mice.

### 2.5 Voluntary exercise elicits sex‐specific effects on cardiac hypertrophy in HFD mice

**FIGURE 5:**
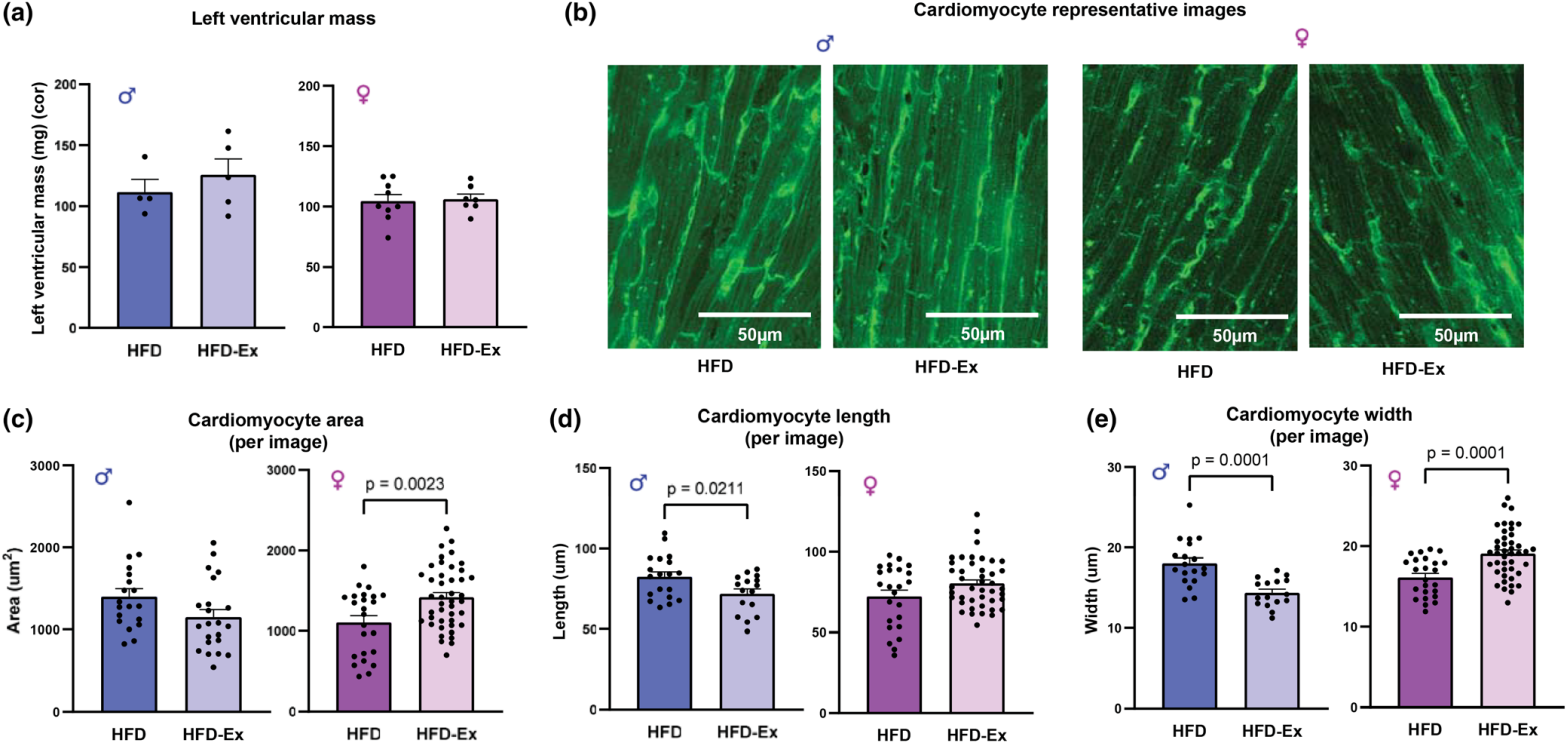
Exercise elicits sex‐specific effects on cardiac hypertrophy in HFD‐fed mice. (a) Left ventricular mass of male and female HFD and HFD‐Ex mice hearts following 10 weeks of HFD feeding. (b) Representative images of cardiomyocytes of male and female HFD and HFD‐Ex mice hearts at 40× oil magnification (c) Cardiomyocyte area (per image) of male and female HFD and HFD‐Ex mice. (d) Cardiomyocyte length (per image) of male and female HFD and HFD‐Ex mice. (e) Cardiomyocyte width (per image) of male and female HFD and HFD‐Ex mice. Data are presented as individual values ± SEM. Data were analyzed by Student's *t*‐test.

Following the observation that exercise improves diastolic function in HFD‐Ex females but not HFD‐Ex males, we then sought to determine if the changes in cardiac function correspond with changes in structure or morphology of the heart. We have previously demonstrated that cardiac hypertrophy is a consistent feature of the HFD mouse model (Daniels et al., 2023). Left ventricular mass was not altered by exercise in either male (*p* = 0.45) or female (*p* = 0.82) mice (Figure 5a); however, total heart weight was greater in both HFD‐Ex male and female mice when adjusted for body weight (Table S1). Surprisingly, exercise elicited differential effects on cardiomyocyte size in males and females. HFD‐Ex females exhibited increased cardiomyocyte area (Figure 5c, *p* = 0.0023) and width (Figure 5e, *p* = 0.0001) when compared with HFD females, indicative of cardiomyocyte hypertrophy. In contrast, HFD‐Ex males exhibited a decrease in cardiomyocyte length (Figure 5d, *p* = 0.021) and width (Figure 5e, *p* = 0.0001) compared with HFD males, suggesting that exercise decreases cardiomyocyte size in males. These data suggest that exercise alters cardiac hypertrophy in response to exercise sex‐specifically in mice on a HFD.

## 3 DISCUSSION

Regular exercise is beneficial for the prevention and treatment of cardiometabolic disease, but it is currently unclear how sex differences impact its efficacy. In this study, we utilized a model of voluntary exercise in mice with HFD‐induced obesity to investigate how exercise elicits cardiometabolic benefit differentially in males and females. Daily running distance was greater in female mice, but the extent of exercise tolerance improvement was similar. In females, but not males, exercise attenuated HFD‐induced body and fat mass gain and improved cardiac diastolic function. Anti‐inflammatory effects of exercise were evident in adipose tissue of both male and female high‐fat‐fed mice, but the specific inflammatory cell types and tissue depots affected were sex‐specific. A slight improvement in insulin sensitivity was observed in exercised males, but not females. Surprisingly, cardiomyocyte size was increased with exercise in female HFD mice and decreased with exercise in male HFD mice. This study provides the first evidence that the glucose handling, adipose and cardiac effects of exercise are differentially elicited in males and females in a cardiometabolic disease setting.

In the present study, voluntary exercise attenuated HFD‐induced weight gain and adiposity in females but not in males. Given that the female mice ran ~2‐fold greater average daily distances on the running wheels than male mice, which is consistent with previous observations that female mice exhibit higher voluntary running wheel activity than males (Bartling et al., 2017), we speculate that energy expenditure may have been increased in female mice. Surprisingly, exercise tolerance was similar in males and females suggesting that male mice may require less training to improve exercise capacity. This finding contrasts with literature reporting that female mice have greater exercise capacity than males (Holcomb et al., 2022; Oydanich et al., 2019) and also with the observation that healthy adult men derive the greatest mortality risk reduction when engaging in 300 min/week of moderate/vigorous physical activity, whereas women only required 140 min/week to achieve a similar mortality risk reduction (Axsom et al., 2024). It could therefore likely be that this improvement in exercise tolerance in males is linked to previous reports of males achieving greater increases in maximal oxygen consumption from exercise training than females (Santisteban et al., 2022). In addition to higher running distances, the female‐specific effect of exercise attenuating HFD‐induced fat mass increase could be partially explained by higher fat utilization in females. Previous studies have shown that female mice display a greater ability to mobilize and use fatty acids as energy during exercise (Holcomb et al., 2022), and in humans, women derive proportionately more energy from fatty acid oxidation during endurance exercise, whereas men derive proportionately more energy from carbohydrate oxidation (Horton et al., 1998). Thus, it is possible that the dietary fat was preferentially utilized as a fuel source in the female mice during exercise, resulting in less fat storage.

It is well established that increased adiposity is closely associated with impaired glucose homeostasis (Appiakannan et al., 2020; Williams et al., 2014). Given the dramatic effect of exercise on reducing fat deposition in HFD female mice, it is surprising that no effect of exercise on blood glucose, glucose tolerance, or insulin tolerance was observed. A trend for improved glucose tolerance was observed in female HFD‐Ex mice, suggesting that the weight loss effect may translate into glycemic benefit with longer exercise duration. On the contrary, male mice had no effect of exercise on body or fat mass but displayed a significant improvement in insulin tolerance. The extent of HFD‐induced impaired glucose homeostasis in the present study has not been determined statistically as control diet comparator groups were not included. Previous studies have established that glucose and insulin intolerance are evident with similar dietary interventions in male and female mice (Garg et al., 2011), although to a lesser extent in females (Casimiro et al., 2021; Pettersson et al., 2012). The exercise regime employed in the present study (voluntary running wheel) is relatively mild and improvements in systemic glucose handling may emerge with longer and/or more intense exercise protocols.

Despite clear sex differences in the effect of exercise on fat deposition, both males and females demonstrated an anti‐inflammatory response to exercise in adipose tissue. However, the cell types and fat depots involved were sex‐specific. HFD‐Ex female mice exhibited expansion in the CD25+ regulatory T cell population and a reduction in the total macrophage population in subcutaneous fat. Female gonadal fat displayed variable changes in pro‐ and anti‐inflammatory cell types with exercise, where increased CD3+, CD4+ and macrophages appeared to be offset by decreased myeloid cells and increased eosinophils. Sex differences in the effect of a HFD on adipose tissue inflammation have been reported previously. Expansion of the pro‐inflammatory M1 macrophages is a key feature of diet‐induced adipose tissue inflammation in male mice, an effect which is not as prominent in female mice (Pettersson et al., 2012). Systemic inflammation and exercise‐induced myokines were not directly measured in the present study. Given the strong immunological responses observed in adipose tissue, and the evidence in the literature that adipose inflammation has a systemic impact, it could be speculated that exercise similarly improved systemic inflammation in HFD mice. The mass of subcutaneous and gonadal fat pads was dramatically lower in HFD‐Ex female mice, and the difference in the immune cell profile observed in HFD mouse adipose tissue with exercise may be directly related to lower adipocyte size and number. In obesity, adipose tissue expansion (adipocyte hypertrophy and proliferation) is an early feature and adipocyte dysfunction is associated with the secretion of inflammatory adipokines. Recruitment of immune cells from the circulation then shifts the balance of pro‐ and anti‐inflammatory cell types (Kawai et al., 2021). In the present study, voluntary exercise partially prevented increased adiposity with HFD in female mice leading to less immune cell recruitment into the adipose tissue.

In obesity, systemic and tissue inflammation is a key feature with a significant impact on the heart. In the present study, we demonstrated that cardiac diastolic function is improved with voluntary exercise in HFD‐fed female mice. This is consistent with a previous study that examined the effect of exercise training in female mice, which reported that voluntary exercise prevented diastolic dysfunction in HFD‐fed females (Bostick et al., 2017). However, exercise was insufficient to improve cardiac function in the HFD‐Ex male mice. It has been reported that female mice with diet‐induced heart failure with preserved ejection fraction (HFpEF) have minimal increases in E/e′ ratios when compared with males (Tong et al., 2019) which may be underpinning our observations. Our observed sex differences in cardiac diastolic function could also be partly explained by the ~2‐fold greater average running wheel distances run by HFD‐Ex females, as there may be a relationship between intensity/duration of exercise and its beneficial effects on diastolic function. In contrast, ejection fraction was slightly lower with exercise in the HFD‐Ex females. Although physiological hypertrophy is a beneficial adaptation that occurs in response to the increased mechanical stress on the heart muscle from exercise training and is generally associated with preserved or enhanced measures of cardiac function (Oláh et al., 2015), some contrasting effects have been reported that exercise is associated with lower left ventricular ejection fraction and end‐systolic volume in a setting of cardiometabolic disease (Fournier et al., 2014). The HFD‐Ex female mice appear to have early signs of cardiomyocyte hypertrophy, which could influence systolic function in this setting. Previous studies have demonstrated that exercise training induces left ventricular physiological hypertrophy in HFD‐fed females but not male mice (Tóth et al., 2022). In the present study, raw heart weights were unchanged, but cardiac weight index (heart weight: body weight) was higher in both HFD‐Ex males and HFD‐Ex females. Female HFD‐Ex mice also displayed larger cardiomyocyte dimensions. Conversely, we observe that HFD‐Ex male mice showed early signs of decreased cardiomyocyte size, suggesting that exercise has the opposite effect in the male mouse heart. This difference between sexes is consistent with literature that female mice display greater exercise‐induced cardiac hypertrophy compared with males, due to enhanced activation of Akt, MAPK, and CaMKII protein kinases that induce cardiac remodeling (Martin & Leinwand, 2024). It is worth noting that the opposite effect has been observed in human studies, with men typically developing greater physiological cardiac hypertrophy in response to exercise training than women (Martin & Leinwand, 2024). This highlights the need for future research into examining human sex differences in cardiac function and structure in response to training in a setting of cardiometabolic disease.

### 3.1 Limitations

In our model, voluntary wheel running was used because it more closely mimics recreational exercise in humans as opposed to forced treadmill running; however, the different running distances between males and females are an important consideration for interpretation of the findings here. As food intake and energy expenditure were not quantified between groups, it cannot be determined whether the primary mechanism underlying the sex differences in fat mass gain in exercised mice was due to metabolic or behavioral changes, or a combination of the two. Additionally, because it has been demonstrated previously that exercise training (endurance and high‐intensity interval training on a treadmill) does reduce fat mass gain and improve glucose homeostasis in male mice (Maharjan et al., 2021), it could be possible that the voluntary running wheel exercise approach was insufficient to observe an effect in these parameters. The present study employed a mouse model of cardiometabolic disease to investigate the effect of exercise. Important differences in the effects of exercise on fuel utilization between mice and humans should be considered when extrapolating these findings to a human context. In general, mice and humans exhibit a similar fuel switch towards lipid utilization with low‐intensity exercise, but mice are relatively more reliant on fatty acid oxidation (lower respiratory exchange ratio) and exhibit a stronger proteolysis response to exercise than humans (Axsom et al., 2024). In response to acute high‐intensity exercise, mice deplete glycogen stores more rapidly thus switching to a greater reliance on gluconeogenesis than humans (Axsom et al., 2024). The translation of the findings from the present study to human responses to exercise requires further investigation.

## 4 CONCLUSIONS

Exercise interventions are becoming increasingly popular as personalized, non‐pharmacological approaches for prevention or treatment of cardiometabolic disease. This study provides evidence that exercise has differential effects on cardiometabolic health in male and female mice in a setting of metabolic disease and provides new insight into sexually dimorphic mechanisms in fat mass expansion, adipose tissue inflammation, cardiac function and structure that underpin the effect of exercise in obese metabolic disease. These findings characterize sex differences in the molecular and cellular basis of exercise benefit in a disease setting. This new knowledge provides an important advance towards personalized exercise interventions and/or towards the development of sex‐specific therapies as exercise mimetics for cardiometabolic disease.

## 5 METHODS

### 5.1 Experimental animals

All animal experiments were approved by the University of Auckland Ethics Committee (AEC Approval Number 2280) and complied with the guidelines and regulations of the Code of Practice for the Care and Use of Animals for Scientific Purposes. C57Bl/6J mice were housed in a temperature‐controlled environment at 22°C with 12‐h light/dark cycles. Upon weaning until 10 weeks of age, animals were fed chow diet (TekLad Madison, WI USA) and water was provided ad libitum. At 10 weeks of age, animals were randomly assigned to experimental groups and were housed two to a cage, with one mouse given access to a running wheel (Columbus Instruments, Columbus, OH USA) ad libitum, and one remaining sedentary. A clear plastic divider with small holes was placed in between the mice to separate them, while still allowing for socialization. High‐fat diet (60% fat by calorie content; SF13‐092; Specialty Feeds, Australia) was provided ad libitum along with water. All in vivo phenotyping was performed once mice had been on the high‐fat diet and exercise intervention for 10 weeks, with a minimum of 48 h recovery time in between each phenotyping test.

### 5.2 Body composition

Body composition (lean and fat mass) of the mice was assessed using an EchoMRI (LLC, Houston, TX, USA) after 10 weeks of HFD intervention.

### 5.3 Exercise

#### 5.3.1 Daily running distance

Each mouse allocated to the exercise intervention group had continuous access (24 h) to a running wheel attached to the cage lid (Model: 0297‐004M, Running Wheel for Mouse Home Cage, Columbus Instruments, OH, USA) (Hwang et al., 2025). Daily wheel revolutions of individual mice were measured using a digital counter attached to each running wheel (Columbus Instruments, Columbus, OH USA) and recorded using CI‐Link software (Columbus Instruments, Columbus, OH USA). Individual distances were then calculated by multiplying revolutions by wheel circumference.

#### 5.3.2 Exercise tolerance test

Exercise tolerance was measured using a motorized treadmill without incline (Panlab/Harvard Apparatus Holliston, MA, USA). Mice were motivated to run by a shock grid at the rear of the belt, and exhaustion time was recorded when mice remained on the shock grid for >3 consecutive seconds. Running capacity was determined as previously described by our group (Watson et al., 2024), by running mice at an initial speed of 10 cm per s, which was increased by 4 cm per s every 3 min until exhaustion.

### 5.4 Glucose and insulin tolerance tests

Insulin (ITT) and glucose (GTT) tolerance tests were performed in 6 h fasted mice, by intraperitoneally injecting a bolus of insulin (Onelink, cat CSP052; 0.75 mU/kg body weight) or oral gavage of D‐glucose (ThermoFisher, cat BSPGL903.500; 1.5 mg/kg body weight) prepared in sterile phosphate‐buffered saline (PBS). Blood samples were collected from tail vein bleeds at time points 0, 15, 30, 45, 60, 90, 120 min post insulin/glucose bolus, and blood glucose was determined using a handheld glucometer (Accu‐chek performa; Roche, Basal, Switzerland).

### 5.5 Echocardiography

Echocardiography was performed following 10 weeks of dietary intervention. The mice were anesthetized with isoflurane (4%, Baxter Healthcare) mixed with oxygen and room air and maintained with 1.5%–2.5% isoflurane (with 1 L/min O_2_) for the duration of the echocardiographic assessment (<45 min per animal). The chest area was shaved prior to the procedure. Transthoracic echocardiography was performed using the VEVO LAZR‐X 3100 with a MX400 (21–44 Hz) linear array transducer coupled to a digital ultrasound system (FUJIFLIM Visual Sonics) as previously described by our group (Daniels et al., 2023). Mice were secured to a warm platform containing ECG lead pads. All analyses were performed by an investigator blinded to sex and exercise, using Vevo LAB software version 5.6.1 (FUJIFLIM Visual Sonics).

#### 5.5.1 M‐mode echocardiography imaging

Left ventricular (LV) M‐mode two‐dimensional echocardiography was performed in a parasternal short‐axis view at the mid‐papillary level to measure LV wall and chamber dimensions. LV volumes were derived from dimensions using the Teichholz equation (end diastolic volume (EDV) = (7/(2.4 + LV internal diameter at end‐diastole (LVIDd))) × LVIDd; end systolic volume (ESV) = (7/(2.4 + LVID at end‐systole (LVIDs))) × LVIDs). Systolic function parameters: % ejection fraction ((EDV – ESV)/EDV × 100), and % fractional shortening ((LVIDd−LVIDs)/(LVIDd) × 100). LV mass was calculated: 1.053 × (LVIDd + LV end‐diastolic posterior wall thickness + end‐diastolic inter‐ventricular septal wall thickness)^3^−LVIDd^3^, with a correction factor of 0.8. At least 3 consecutive cardiac cycles were sampled per cineloop image and 3 images per animal were analyzed and averaged.

#### 5.5.2 Pulse wave doppler and tissue doppler imaging

Pulse wave Doppler and tissue Doppler imaging were acquired from the apical 4 chamber view to assess LV diastolic function parameters: velocity of mitral inflow during early passive filling (E) and velocity of mitral annulus during early passive filling (e′) and E‐wave deceleration time. At least 3 cardiac cycles were sampled per cineloop image and 2–4 images per animal were analyzed and averaged.

### 5.6 Tissue collection

Mice were euthanised via CO_2_ inhalation and tissues were rapidly dissected and weighed. Hearts were washed in Kreb's heart buffer (146 mM NaCl, 4.69 mM KCl, 0.35 mM NaH_2_PO_4_, 1.05 mM MgSO_4_, 10 mM HEPES, 11.1 mM D‐Glucose, and pH 7.40) and cardiac midsections were dissected and fixed in 4% of paraformaldehyde (Sigma, cat – 1.00496). Subcutaneous and epididymal fat pads were collected in cold PBS.

### 5.7 Flow cytometry

To isolate the stromal vascular fraction, visceral (gonadal), and subcutaneous fat pads were excised and chopped into fine pieces in 2 mL of PBS, incubated in 6 mL of digestion buffer (2 mg collagenase B (#17101015, Thermo Fisher Scientific, USA), 0.1 mM CaCl, 0.5% bovine serum albumin (#ABRE, MP Biomedicals, USA), 0.1 mg DNAase (#DN25, Sigma Aldrich, USA) in PBS) for 60 min at 37°C, 250 rpm, and washed through a 100 μm filter with FACS buffer (PBS, 2% calf serum, 0.25 M EDTA). The suspension was pelleted by centrifugation (500 g, 10 mins, 4°C) and the resulting cell pellet was lysed in red blood cell lysing buffer (0.15 M NH_4_Cl, 1.0 mM NaHCO_3_, and 1.27 mM EDTA) for 5 min to remove residual red blood cells, and washed twice in FACS buffer by centrifuging at 300 *g* for 5 min. The immune cell‐enriched pellet was resuspended in 50 μL of FACS buffer containing antibodies (Table S3) and incubated on ice for 30 min (light protected). Excess unbound antibody was removed by washing twice in FACS buffer and centrifuging at 300 *g* for 5 min. The final pellet was then suspended in FACS buffer before being analyzed on a Northern Lights Flow Cytometer (Cytek Biosciences, Fremont, CA USA). Gating strategy is outlined in Figure S2. Data were analyzed using FlowJo V 10.4.2.

### 5.8 Histology

Cardiac midsections were fixed in 4% paraformaldehyde (Sigma, cat – 1.00496) and then cryopreserved by incubating in 10% sucrose, then 20% sucrose, and then in 30% sucrose overnight prior to optimal cutting temperature compound (OCT) embedding in precooled isopentane (Sigma, cat—M32631‐500ML). Sections were cut (10 μm) and stained with Wheat Germ Agglutinin—Alexa Fluor™ 594 (WGA‐AF594) conjugate 1:100 (Sigma, cat—W11262). Tissue sections were imaged using a Zeiss LSM 710 inverted confocal microscope under 40× oil magnification, with image capture software Zeiss ZEN Black (Carl Zeiss Microscopy, USA). Cardiomyocyte dimensions were traced using ImageJ software (NIH, Bethesda, USA), as shown in Figure S3.

### 5.9 Statistical analysis

Statistical analysis was performed using Prism v.8.0.0 (GraphPad Software Incorporated San Diego, CA, USA) with statistical significance determined as *p* < 0.05 and checked for normal distribution where appropriate using Shapiro Wilk's test. Specific statistical tests used are indicated in the figure legends. Data are presented as mean ± standard error of the mean (SEM). SEM (rather than standard deviation) has been used in this study to reflect the precision of the sample mean as an estimate of the population mean, thus providing an indication of the reproducibility of the data given the relatively small sample size employed.

## AUTHOR CONTRIBUTIONS

L.E.W., T.L.M., and K.M.M. conceived and designed the research. L.E.W., M.A., C.L.M., R.F.D., and C.P.H. performed animal monitoring and in vivo experiments. L.E.W, J.D., N.B., R.F.D., and T.C. performed molecular experiments. L.E.W., T.L.M, K.M.M., R.F.D., C.P.H, M.A., and C.P. interpreted results of experiments. L.E.W., K.M.M., and T.L.M. drafted the manuscript. All authors approved the final version.

## CONFLICT OF INTEREST STATEMENT

The authors declare that they have no competing interests.

## ETHICS STATEMENT

All animal experiments were approved by the University of Auckland Ethics Committee (AEC Approval Number 2280) and complied with the guidelines and regulations of the Code of Practice for the Care and Use of Animals for Scientific Purposes.

## Supporting information


Table S1.

Table S2.

Table S3.

Figures S1–S3.

## Data Availability

The datasets generated and analyzed during the current study are available from the corresponding author on reasonable request.
