# Supplementary material for: Exercise has differential cardiometabolic effects in male and female mice on a high‐fat diet
Source: Physiol Rep. 2026 Jan 28;14(2):e70656. doi: 10.14814/phy2.70656 (PMC12848585; doi:10.14814/phy2.70656)
Supplement: Supplementary file 1 — Table S1. [file PHY2-14-e70656-s003.docx]

**Supplementary Table S1. Cardiac characteristics.**

|  | **Male** | | **Female** | |
| --- | --- | --- | --- | --- |
|  | **HFD** | **HFD-Ex** | **HFD** | **HFD-Ex** |
| **Cardiac weight index (mg/g body weight)** | 3.42 ± 0.09 | 3.83 ± 0.13* | 3.68 ± 0.09 | 4.06 ± 0.16* |
| **Cardiac output (mL/min)** | 22.5 ± 1.2 | 24.4 ± 1.7 | 20.3 ± 1.0 | 20.1 ± 0.6 |
| **Heart rate**  **(beats per minute)** | 516 ± 16 | 547 ± 5 | 526 ± 9 | 520 ± 8 |

Cardiac weight index: total heart weight (mg) standardised to body weight (g. Data are presented as mean values ± SEM. Data were analysed by Student’s T-test. Significance was determined at *p<0.05.
