# Supplementary material for: Exercise has differential cardiometabolic effects in male and female mice on a high‐fat diet
Source: Physiol Rep. 2026 Jan 28;14(2):e70656. doi: 10.14814/phy2.70656 (PMC12848585; doi:10.14814/phy2.70656)
Supplement: Supplementary file 2 — Table S2. [file PHY2-14-e70656-s001.docx]

**Supplementary Table S2. Weight and body composition of HFD and HFD-Ex mice.**

|  | **Male** | | **Female** | |
| --- | --- | --- | --- | --- |
|  | **HFD** | **HFD-Ex** | **HFD** | **HFD-Ex** |
| **Starting BW (g)** | 27.1 ± 0.5 | 27.6 ± 0.5 | 21.2 ± 0.6 | 20.4 ± 0.7 |
| **End-point BW (g)** | 45.8 ± 1.5 | 42.9 ± 1.3 | 41.2 ± 1.4 | 34.5 ± 1.3** |
| **Fat mass (g)** | 20.6 ± 2.8 | 16.5 ± 1.2 | 14.8 ± 0.9 | 9.8 ± 1.1** |
| **Lean mass (g)** | 26.7 ± 0.8 | 29.3 ± 0.7* | 21.7 ± 0.9 | 20.7 ± 0.6 |

Data are presented as mean ± SEM. Data was analysed by Student’s T-test. Significance was determined at * p<0.05 and **p<0.01
