# Supplementary material for: Exercise has differential cardiometabolic effects in male and female mice on a high‐fat diet
Source: Physiol Rep. 2026 Jan 28;14(2):e70656. doi: 10.14814/phy2.70656 (PMC12848585; doi:10.14814/phy2.70656)
Supplement: Supplementary file 3 — Table S3. [file PHY2-14-e70656-s002.docx]

**Supplementary Table S3**: Flow Cytometry Antibodies

| **Antigen** | **Fluorophore** | **Clone** | **Supplier** | **Cat** |
| --- | --- | --- | --- | --- |
| CD45 | AF532 | 30-F11 | ThermoFisher | 58-0451-80 |
| CD11b | AF700 | M1/70 | BioLegend | 101222 |
| CD64 | APC | FcγRI | BioLegend | 139305 |
| CD3 | APC-fire 750 | 17A2 | BioLegend | 100248 |
| IA/IE (MHCII) | BV480 | 2G9 | BD Biosciences | 746669 |
| CD8 | BV570 | 53-6.7 | BioLegend | 100740 |
| CD206 | BV605 | C068C2 | BioLegend | 141721 |
| LY6G | BV650 | 1A8 | BioLegend | 127641 |
| CD11c | BV785 | N418 | BioLegend | 117335 |
| CD4 | Pacific Blue | GK1.5 | BioLegend | 100427 |
| F480 | PE-Dazzle594 | BM8 | BioLegend | 123145 |
| CD24 | PE-Cy5 | M1/69 | BioLegend | 101811 |
| CD25 | PE-Cy7 | 3C7 | BioLegend | 101915 |
