# Supplementary material for: Exercise has differential cardiometabolic effects in male and female mice on a high‐fat diet
Source: Physiol Rep. 2026 Jan 28;14(2):e70656. doi: 10.14814/phy2.70656 (PMC12848585; doi:10.14814/phy2.70656)
Supplement: Supplementary file 4 — Figures S1–S3. [file PHY2-14-e70656-s004.pdf]

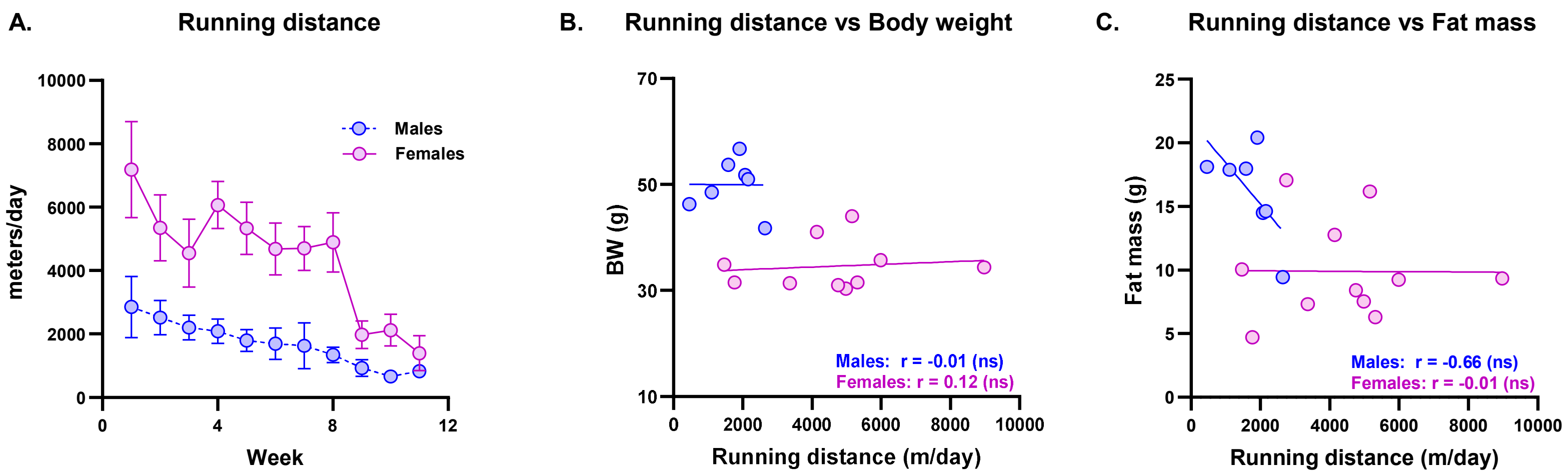

Supplementary figure S1. Timecourse of running wheel distance data for male and female HFD mice (A) and correlations with body weight (B) and fat mass (C). Data presented as mean +/- SEM (panel A). r, Pearson's correlation coefficient; ns, not statistically significant.

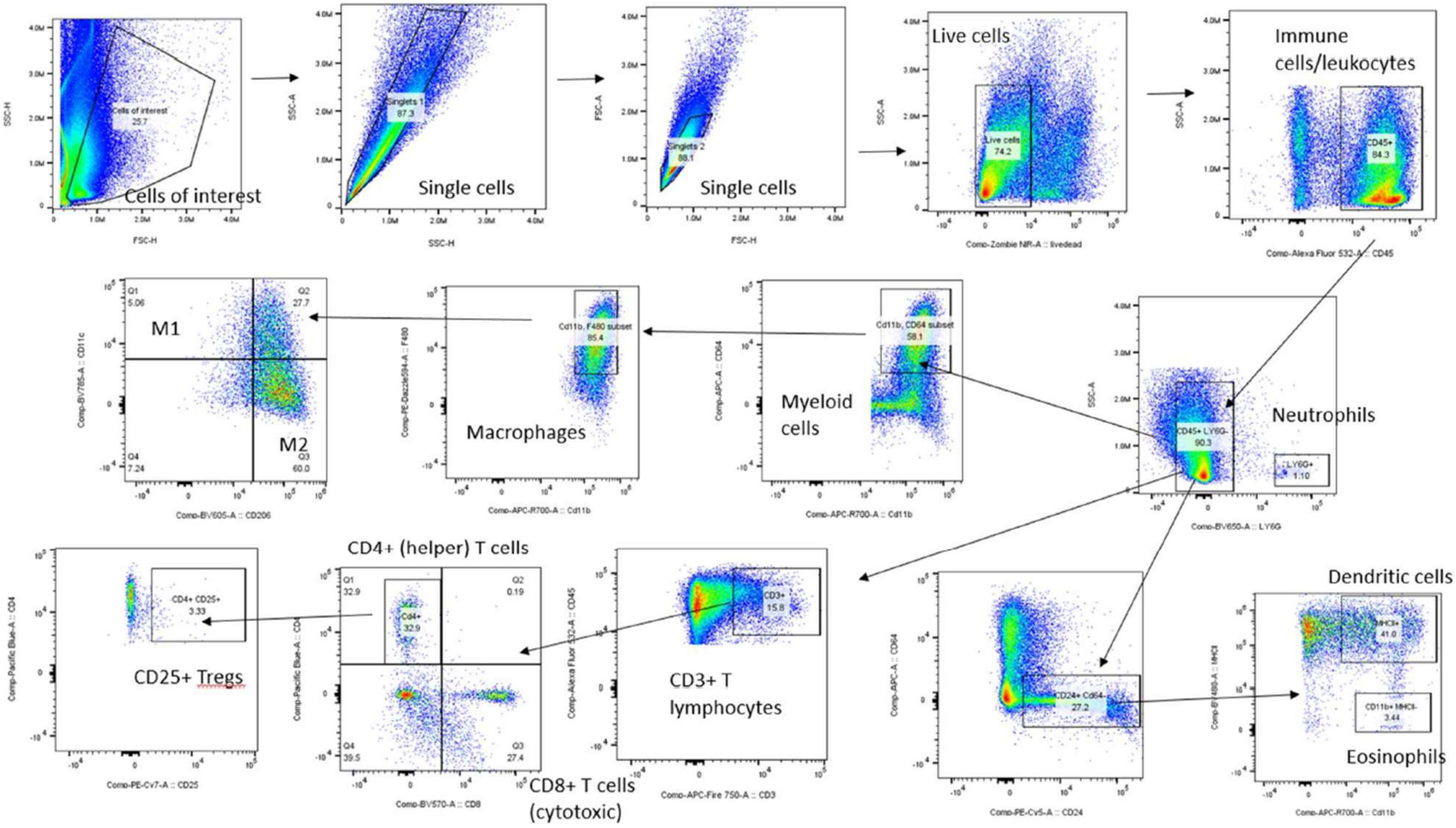

Supplementary Figure S2: Gating strategy for fluorescent-activated cell sorting. Data were analysed using FlowJo software V 10.4.2.

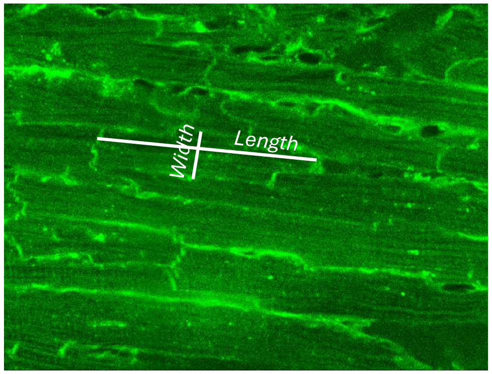

Supplementary figure S3. Exemplar WGA-stained cardiac tissue showing measurements of maximum length and maximum width.
